# Commercial SARS-CoV-2 Targeted, Protease Inhibitor Focused and Protein–Protein Interaction Inhibitor Focused Molecular Libraries for Virtual Screening and Drug Design

**DOI:** 10.3390/ijms23010393

**Published:** 2021-12-30

**Authors:** Sebastjan Kralj, Marko Jukič, Urban Bren

**Affiliations:** 1 Laboratory of Physical Chemistry and Chemical Thermodynamics, Faculty of Chemistry and Chemical Engineering, University of Maribor, Smetanova 17, SI-2000 Maribor, Slovenia; sebastjan.kralj1@um.si (S.K.); marko.jukic@um.si (M.J.); 2 Faculty of Mathematics, Natural Sciences and Information Technologies, University of Primorska, Glagoljaška 8, SI-6000 Koper, Slovenia

**Keywords:** targeted libraries, focused libraries, computer-aided drug design, virtual screening, in silico drug design, high-throughput virtual screening

## Abstract

Since December 2019, the new SARS-CoV-2-related COVID-19 disease has caused a global pandemic and shut down the public life worldwide. Several proteins have emerged as potential therapeutic targets for drug development, and we sought out to review the commercially available and marketed SARS-CoV-2-targeted libraries ready for high-throughput virtual screening (HTVS). We evaluated the SARS-CoV-2-targeted, protease-inhibitor-focused and protein–protein-interaction-inhibitor-focused libraries to gain a better understanding of how these libraries were designed. The most common were ligand- and structure-based approaches, along with various filtering steps, using molecular descriptors. Often, these methods were combined to obtain the final library. We recognized the abundance of targeted libraries offered and complimented by the inclusion of analytical data; however, serious concerns had to be raised. Namely, vendors lack the information on the library design and the references to the primary literature. Few references to active compounds were also provided when using the ligand-based design and usually only protein classes or a general panel of targets were listed, along with a general reference to the methods, such as molecular docking for the structure-based design. No receptor data, docking protocols or even references to the applied molecular docking software (or other HTVS software), and no pharmacophore or filter design details were given. No detailed functional group or chemical space analyses were reported, and no specific orientation of the libraries toward the design of covalent or noncovalent inhibitors could be observed. All libraries contained pan-assay interference compounds (PAINS), rapid elimination of swill compounds (REOS) and aggregators, as well as focused on the drug-like model, with the majority of compounds possessing their molecular mass around 500 g/mol. These facts do not bode well for the use of the reviewed libraries in drug design and lend themselves to commercial drug companies to focus on and improve.

## 1. Introduction

**Figure 1:**
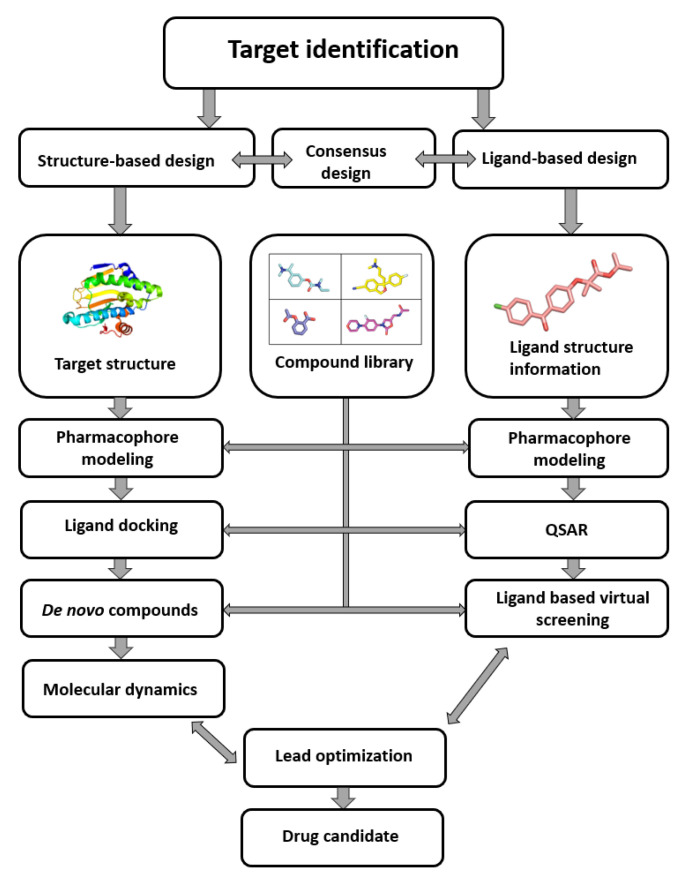
Computer-aided drug design process chart used to obtain lead compounds (based on Sliwoski et al. [5]).

After the identification of a biological target (enzyme, receptor, protein, etc.), the focus of the early phase of drug discovery rests on the identification of leads or compounds that exhibit pharmacological activity against this specific target [1,2]. This represents the first step in the lengthy process of drug discovery, which includes the phases of target to hit, hit to lead, lead optimization, clinical trials and market launch, followed by the phase four [3]. The process of discovering new drugs based on the knowledge of their biological target is called drug design, where the final goal is a molecule complementary in shape and charge to its target and with a desired pharmacological profile [4]. Computer-aided methods have become increasingly important over the years to the extent that they are indispensable in modern drug development. Computer-aided drug design (CADD) generally follows three main objectives: (1) filtering large compound libraries into smaller groups of prospective compounds, (2) optimization of lead compounds and (3) development of novel compounds [5,6]. The term “CADD” is also used for two general approaches, e.g., structure-based and ligand-based drug design (Figure 1). As the name suggests, the structure-based drug design relies on the knowledge of the target structure, while the ligand-based drug design applies the known active and inactive compounds through similarity search, quantitative structure–activity relation (QSAR) studies or through the application of modern machine-learning techniques that are becoming increasingly important in the field [7,8,9].

The use of CADD reduces the costs and labor associated with the drug development, but as can be seen in Figure 1, the success of this process depends on the richness of the initial compound library. Due to the sheer size of the entire chemical space (estimates for the number of synthesizable organic compounds vary from 10^30^ to 10^60^), it is impossible to search it in its entirety. Consequently, extensive compound libraries representing the appropriate chemical space are critical for the success of (virtual) high-throughput screening (vHTS or HTS) [10].

This is a computational methodology used to differentiate between the molecules from a virtual library that bind to a potential target and those that do not, as well as to rank these molecules according to their predicted binding strength. This allows us to enrich the hit rate (the number of binding compounds at a given concentration divided by the number of compounds tested experimentally) over classical screening methods, such as the high-throughput screening (HTS) [11]. The popularity of the method is increasing with the larger availability of structural information, such as 3D protein structures, and with the commercially available libraries. The two factors work together in a tandem to provide a broad platform for vHTS [12]. Compared to the classical HTS, which is still used today as a staple for confirming activity against a target, vHTS offers the ability to pre-process enormous lists of molecules, reducing the number of compounds that need to be purchased, synthesized, and tested [13,14]. For example, the researchers at Pfizer used a vHTS docking protocol to screen for inhibitors of the enzyme tyrosine phosphatase 1B. The vHTS protocol yielded 365 compounds, of which 127 exhibited effective inhibition. Compared to a classical HTS protocol performed in parallel with the vHTS, the hit count for the 400,000 compounds tested was 81, representing a hit rate of only 0.021%, compared to 34.7% in the vHTS protocol [11]. Moreover, the method yields structurally diverse lead compounds and can be extended to compounds that do not yet physically exist but can be obtained by synthesis or that serve as fragments in the later stages of the development [2,15,16,17]. Virtual screening or high-throughput virtual screening can be performed by using one or a combination of QSAR, pharmacophore-based virtual screening (PBVS), molecular docking (docking-based virtual screening, DVBS) and modern machine learning techniques [18,19,20,21,22,23,24,25]. What is crucial and common to all approaches is that extra care should be taken in regard to the input data, e.g., the virtual compound libraries.

Pharmaceutical companies have realized the importance of possessing chemical libraries that cover a vast and diverse chemical space. Consequently, They have created large libraries of several million compounds (Pfizer, 4 million [26]; Novartis, 1.7 million [27]; and Astra Zeneca, 4 million, accessible through collaborations [28]). To date, the main method for searching for lead molecules in large compound libraries remains HTS [29]. HTS, the experimental counterpart of vHTS, identifies lead molecules by examining individual biochemical assays for each of the more than one million molecules in the respective library [13]. The main challenge of HTS is to find a balance between the size of the library, its structural diversity and the cost of screening the compounds [30]. From this perspective, the modern HTS has reached the “plateau of productivity” according to the Gartner Hype Cycle, leading to the integration of fragment screening and computational methods into the process [31]. Several computational methods can be synergistically integrated into the screening process to mitigate the weaknesses of the remaining methods and to increase the hit rate [26]. One finds several reports of drugs discovered by using computational methods with imatinib, a tyrosine kinase inhibitor, as a prime example [32,33].

The main difference between computational methods and HTS represents the use of virtual compound libraries in the process. While physical compound libraries reach the number of millions of molecules, virtual compound libraries created by large pharmaceutical companies can range from 10^7^ to 10^18^ molecules. Investigations of these libraries identify specific molecules, synthetic pathways and focus the research toward a specific chemical space [34,35,36,37,38,39,40,41,42]. As the CADD methodology is nowadays routinely used in drug discovery, the need for virtual compound libraries increases. Consequently, numerous commercial vendors have emerged, offering such libraries in both physical and virtual forms. To further facilitate the drug discovery, commercial vendors are now offering scientists the so-called targeted or focused compound libraries ready to jumpstart the drug design and discovery in a specific field. These focused libraries often represent a subset of compounds from the manufacturer’s full database that may possess specific properties for a selected target or drug design field. This review explores this topic as the first of its kind for the benefit of the medicinal chemistry community and refers the reader to the relevant resources and related reports in the scientific literature.

## 2. In Silico Library Design for Medicinal Chemistry

**Figure 2:**
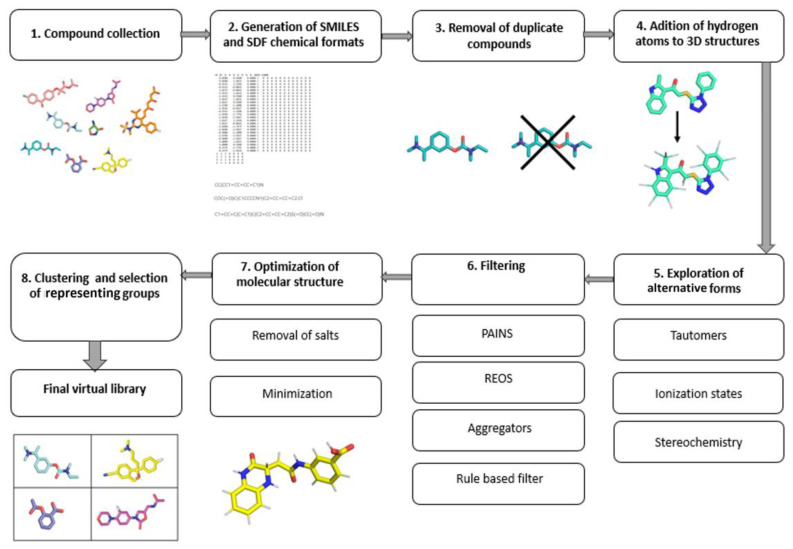
Workflow of an efficient library preparation for medicinal chemistry.

When designing or examining an existing molecular library, a researcher should be aware of important steps and drawbacks associated with virtual molecules. The following chapter addresses these issues and helps the reader to develop both a critical view on the existing libraries, as well as provide a good foundation to create his/her own virtual libraries similar to the one presented in Figure 2 [43,44,45].

After obtaining the target structure and learning about the molecular and biological context, a researcher can proceed toward designing the virtual compound library. Pharmacokinetics and pharmacodynamics represent important aspects to consider when preparing any molecular library. This was discussed by Lipinski et al., who postulated that several drug classes, such as antibiotics, antifungals and vitamins, among others, fell outside the Ro5, due to transporter effects [46]. The term “metabolite-likeness” is also used to name the approach where, instead of similarity to known drugs, the library is constructed by the comparison to known metabolites, an approach that factors in pharmacokinetics [47]. All in all, both are important in molecular library design, as compound structures are critical for their behavior in a complex biological system, where absorption, distribution, metabolism and elimination (pharmacokinetics) are at play [48]. When considering the mode of action of the compound on a relevant target, one should consider all possible interactions formed between the target and the ligand, as well as factor in these data in the library design, if possible. Key interactions from covalent, ionic, metallic to the hydrogen bonds and various dipole-dipole, as well as hydrophobic interactions, should all be considered, and the potential for their formation should be assessed in the constructed compound library.

The main goal of targeted libraries is to cover a diverse chemical space with as few compounds as possible. Due to the fast-growing availability of the chemical and biological structural data, the public bioactivity databases provide an excellent starting point [49]. The most comprehensive and curated information about molecules is freely available in the prominent PubChem [50], ChEMBL [51] and ZINC [52] databases. After assembling the initial database from various sources, the next step is to remove duplicates, so that only unique structures are processed further [49]. The choice of the chemical file format is vital, as it dictates how the obtained data can be used. The most recommended file formats for representing molecules as strings include the SMILES and InChI formats. In most cases, multiple SMILES strings can equally well represent a single molecule. The application of the canonical SMILES, which uses only a single string per molecule, is recommended to avoid duplication and filtering problems. For the spatial representation of molecules, either the Structure Data Format (SDF) or the MDL Molfile (MOL) format are the most common [53]. Online libraries can usually be downloaded in these formats, making it easier to obtain a coherent library. When performing filtering, clustering or similarity searches, the SMILES format is preferred, as it leads to faster processing, due to its string representation; however, the spatial information is required for downstream methods such as 3D pharmacophore development, molecular docking and molecular dynamics. The molecular representation always requires the extra care and exploration in terms of conformational viability, chiral centers, tautomerism, compound ionization, presence of salts, structural faults, etc. By default, the hydrogen atoms are often not present in various chemical file formats and should be added during the library preparation. Tautomerism represents a property of chemical compounds that affects the calculation of their physiochemical properties, such as logP, logD and pKa, and subsequently bears consequences in both QSAR and molecular docking [54]. Due to their different structural representations, tautomers are often handled as separate molecules by CADD programs [49,55]. Moreover, since proteins are known to be enantioselective toward the binding ligands, exploring chirality when designing a virtual library is an important aspect to consider. In the majority of virtual libraries, however, compounds are represented by a single stereoisomer, or the stereo information is absent altogether. Exploring chirality thoroughly will expand the database size by 2^n^ per molecule, where n is the number of chiral centers present. With larger databases, this issue will be even more pronounced and should be considered before generating all possible stereoisomers [56,57]. In general, it is recommended to explore unspecified chirality, which should be performed on a case-by-case basis with regard to the biological context [49]. Furthermore, for compounds that have ionizable groups, multiple different structural representations should be used. Within a reasonable pH range, structures should be represented as protonated and deprotonated forms of compounds [56]. The biological context of the target protein should be used to provide an accurate representation of the environment. After the final compound 3D structure generation, energy minimization should be carried out in order to optimize the molecular geometry [19,58].

Library design should factor in the avoidance of toxic outcomes. Despite the fact that toxicity of drugs is multifactorial and that predicting the exact property responsible for toxicity is difficult, several correlations of toxicity to in vitro pharmacology profiles exist and can be translated to in silico tools which examine molecular descriptors and filter the libraries accordingly [59]. The filtering itself can also flag compounds with reactive functional groups or moieties, optically interfering components, aggregators or frequent hitters. The filtering of “unwanted” molecular species using computational filters represents an essential element of library preparation which should always be considered in a suitable context [60,61]. The essential filters in medicinal chemistry library design are presented in Table 1 below. The size of the chemical space used in CADD means that further entries can be calculated for compounds in molecular libraries, such as structural keys or (hashed) molecular fingerprints. These additional compound representations alleviate steps in the downstream calculations, e.g., clustering and the chemical space definition [62,63].

## 3. Methods

Articles in this review were obtained by searching for keywords (https://pubmed.ncbi.nlm.nih.gov/; accessed on 23 December 2021) related to the formation of chemical libraries, filtering of libraries, methodology in in silico drug design and by following citations provided in other review articles. To obtain the commercial libraries for the assessment, we performed online searches. The keywords for the libraries were as follows: “SARS-CoV-2 targeted molecular library” and “COVID-19 targeted molecular library” for the SARS-CoV-2-targeted libraries; “protein inhibitor targeted molecular library” for the protein-inhibitor-targeted libraries; and “protein–protein interaction targeted library” and “PPI inhibitor targeted molecular library” for the protein–protein-interaction-inhibitor-targeted library. We cross-checked the results with the ZINC database [52]. We proceeded to collect all available structural data, either by directly contacting the vendor or downloading them from the vendor web sources. The data on the assembly of the commercial library were used for the critical analysis and comparison with the general practice. The structural files obtained were subjected to filtering and statistical analysis of descriptors that bear the most information on the chemical space of the library [80].

PAINS, REOS and Lipinski Ro5 were the most common filters that were used by commercial vendors when preparing the libraries, and were as such chosen for testing the quality of the investigated commercial libraries. We additionally applied the aggregator filters often overlooked when preparing molecular libraries. In-house workflows for filtering the obtained libraries filter were created by using Konstanz Information Miner (KNIME, version 4.2.3; http://knime.org; accessed on 23 December 2021). All medicinal chemistry filter KNIME implementations can be found at https://gitlab.com/Jukic/knime_medchem_filters (accessed on 23 December 2021). The results obtained were statistically analyzed and visualized by using KNIME.

## 4. Examples of Commercial Targeted Libraries

We set out to collect the commercially available targeted libraries and to evaluate their potential for drug discovery in the area of SARS-CoV-2. Since targeted libraries use a variety of different methods for data collection (ligand-based, structure-based and various filters), we attempted to gain a better understanding of how libraries are designed and what they offer to the virtual screening process. All links to the libraries collected are provided in the Supplementary Materials of this article. In addition, a more comprehensive list of other commercial libraries, with additional information on the types of compounds that are not a part of this investigation, is provided to guide readers in their drug design efforts.

### 4.1. SARS-CoV-2- or COVID-19-Targeted Libraries

Since December 2019, COVID-19 has caused a global pandemic and shut down public life worldwide. The pressure on the scientific community to develop a vaccine or drug has never been greater [81]. Several SARS-CoV-2 proteins have emerged as potential therapeutic targets for drug development, for example, S-protein and the viral proteases 3CL^pro^ and PL^pro^ [25,82,83,84,85]. With the new potential SARS-CoV-2 targets in mind, we decided to evaluate the commercially available SARS-CoV-2-targeted libraries. Protease-inhibitor-focused and protein–protein-interaction-inhibitor-focused libraries employed in the SARS-CoV-2 drug design were examined, as well. 

#### 4.1.1. Enamine

**Figure 3:**
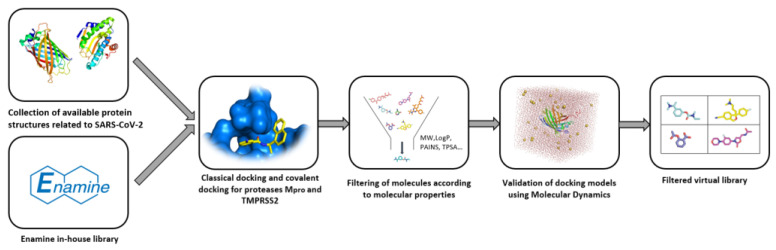
Process algorithm for generating the Enamine SARS-CoV-2-targeted molecular library.

The Enamine library is composed of several different libraries and consists of compounds associated with targets of the SARS CoV-2 virus. The library construction began with the collection of structural data for selected target proteins. Docking models were created and validated by using short molecular dynamics simulations. Covalent docking was performed on cysteine and serine proteases to identify potential covalent binders. Finally, the obtained molecules were filtered by using various medicinal chemistry parameters not disclosed by the commercial supplier (Figure 3).

#### 4.1.2. Otava

The SARS-CoV-2-targeted library supplied by Otava contains eight different sub-libraries. Apart from a single general library, the remaining libraries are targeted against SARS-CoV-2 proteins. The targeted libraries were designed by receptor-based virtual screening, using crystal structures of the target proteins. The entire procedure involved flexible docking of the Otava Drug-Like Green Collection (collection of compounds that satisfy Lipinski’s rule of five) to key binding sites. The relevant protein binding site with the docked molecule was examined in detail. The final decision was based on the structural determinants of ligand binding, docking scores and intermolecular hydrogen bonding within the binding site.

**Figure 4:**
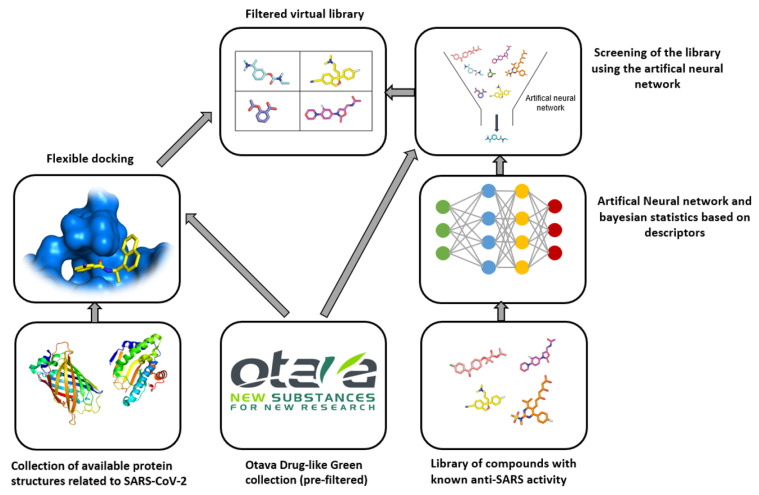
Process algorithm for generating the Otava ltd. SARS-CoV-2-targeted molecular library.

The next library was constructed by using machine learning techniques to obtain molecules with predicted activity against SARS-CoV-2. Initially, the molecules showing activity against coronavirus targets and the inactive compounds were divided into two equal groups. One was used as a training group and the other as a test group. The model based on Bayesian statistics and artificial neural networks was not further disclosed by the supplier nor was the relevant reference to the primary literature provided. The test sets used to validate models and based on a variety of molecular descriptors, such as Molecular Weight, number of hydrogen bond acceptors, number of rotatable bonds, LogP and the polar surface area of molecules. The Drug-Like Green collection was checked against the model, and the highest scoring compounds were visually inspected (Figure 4).

#### 4.1.3. Chembridge

**Figure 5:**
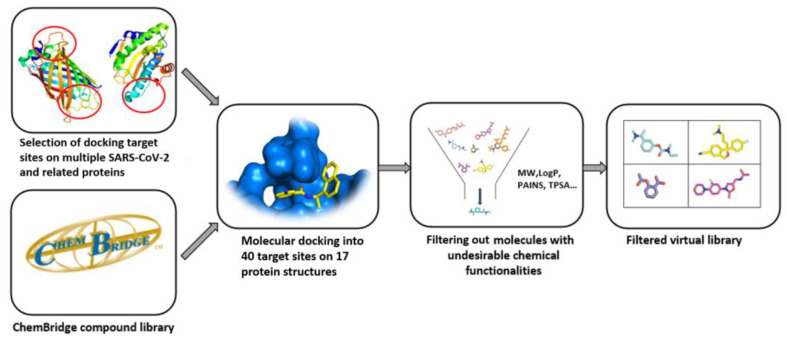
Process algorithm for generating the ChemBridge SARS-CoV-2-targeted molecular library.

This database was constructed in collaboration with academia and contains molecules with potential activity against 17 SARS-CoV-2 targets. The entire Chembridge library was prepared by using VirtualFlow and docked at 40 different sites on 17 protein targets. Over 1.3 million molecules were docked into the targets, which were prepared by using AutoDock tools to transform PDB into PDBQT format. Molecular docking was performed by using QuickVina, and the proteins were held rigid. The best hits were then further filtered to assure lead-like properties (Figure 5) [86]. 

#### 4.1.4. Life Chemicals

**Figure 6:**
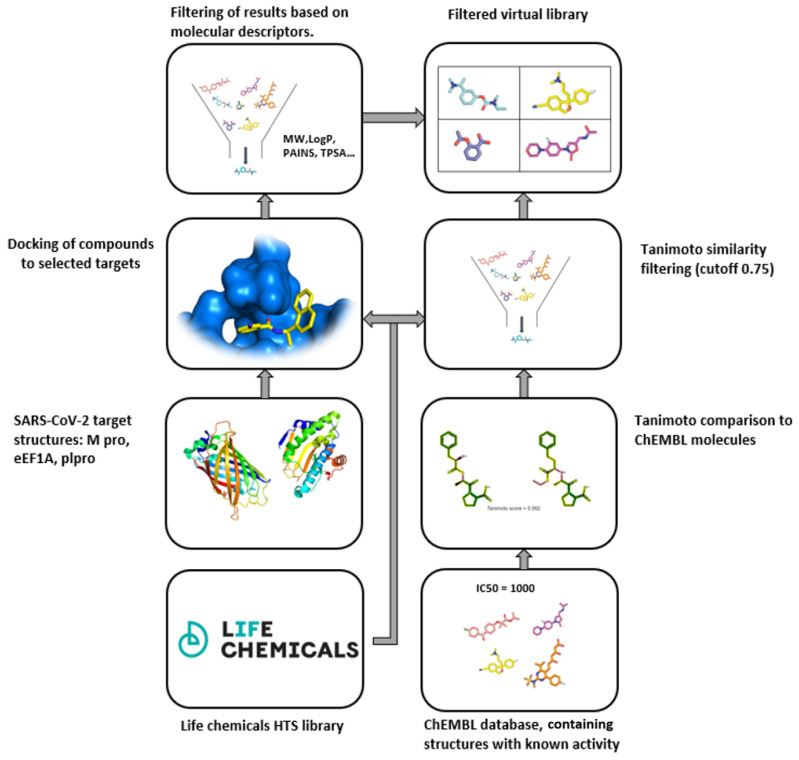
Process algorithm for generating the LifeChemicals SARS-CoV-2-targeted molecular library.

Using docking-based virtual screening, the entire collection was screened against three different coronavirus-associated proteins, using the Glide software. The compounds were further filtered by using Lipinski’s rule of five, with the exception of the main protease, as it would filter out many peptide-like compounds. All molecules in the final database contain no PAINS, toxic or reactive groups. The second part of the library was assembled by using a 2D fingerprint similarity approach. The Tanimoto cutoff was set at 75% for screening molecules with known activity against the SARS coronavirus. Data on active molecules were obtained from ChEMBL. Compounds were selected based on the minimum accepted activity value (IC_50_, K_i_; according to ChEMBL; Figure 6).

#### 4.1.5. TargetMol

**Figure 7:**
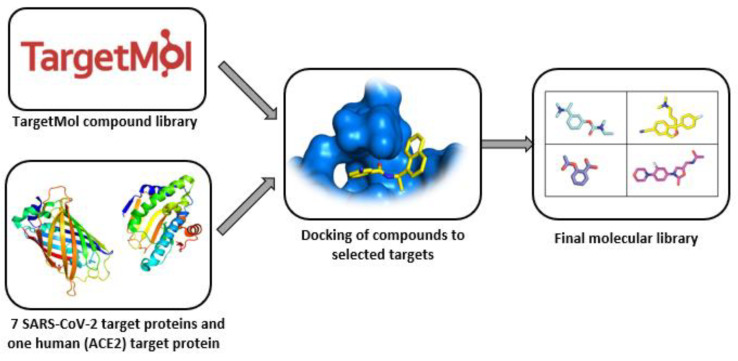
Process algorithm for generating the TargetMol SARS-CoV-2-targeted molecular library.

The library contains compounds shown to be active against coronaviruses or possessing a broad-spectrum antiviral activity. The molecules were obtained by molecular docking screening against seven SARS-CoV-2 target proteins and one human (ACE2) target protein. The library was extended by virtual screening of the natural products obtained from traditional Chinese medicinal plants (Figure 7).

**Figure 8:**
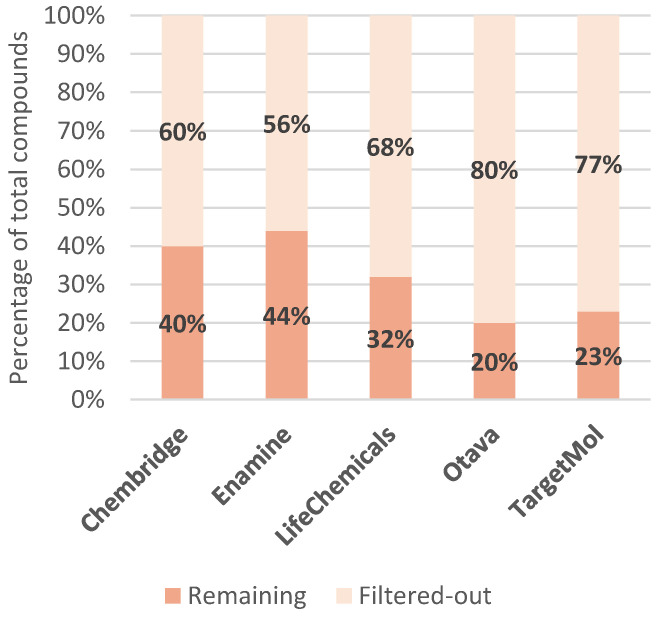
Compound (relative) retention after post-filtering for REOS, PAINS, Aggregators and the Lipinski rule of 5 (one rule break allowed) on protein–protein-interaction-inhibitor-targeted libraries.

For each library described, we performed an analysis of the most common molecular descriptors that were found to be relevant [80]: Molecular Weight (MW), Total Polar Surface Area (TPSA), SlogP, number of hydrogen bond acceptors (HBA), number of hydrogen bond donors (HBD), number of rings present and the number of rotatable bonds (Table 2). Compound retention when passing through each individual filter was assessed, as well (Table 3), with the results for joined filter provided in Figure 8.

### 4.2. Protease-Inhibitor-Focused Libraries

**Figure 9:**
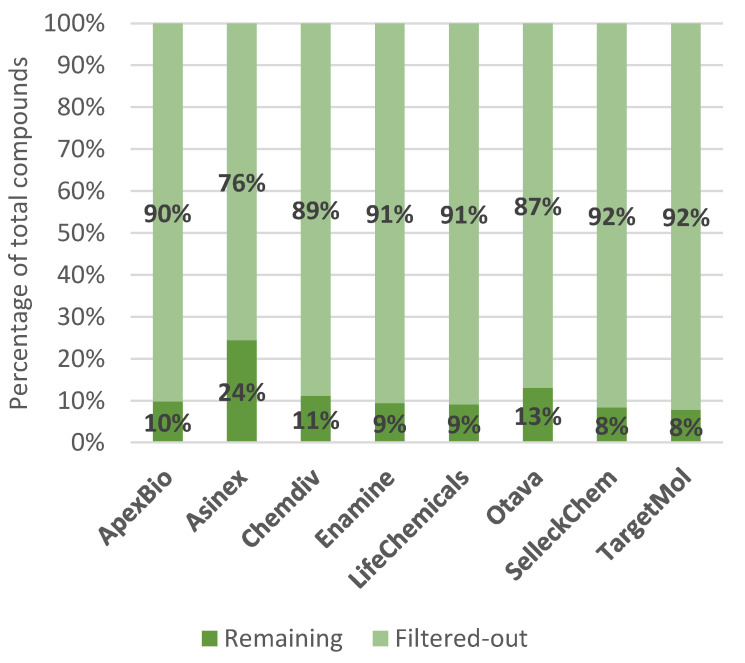
Compound (relative) retention after post-filtering for REOS, PAINS, Aggregators and the Lipinski rule of 5 (one rule break allowed) on protease-inhibitor-targeted libraries.

Proteases are enzymes which break down proteins by the hydrolysis of peptide bonds and are involved in various processes, making them potential targets for drug design [87]. Based on the mechanism of catalysis, several types exist, with the most common being the serine and cysteine proteases [87]. When inspecting such libraries, it is important to note that the design of covalent inhibitors requires different CADD approaches than for the design of non-covalent inhibitors [88]. The TargetMol molecular library consists of 295 protease inhibitors and includes known actives against serine, cysteine, aspartic and other proteases. All compounds have a confirmed IC_50_, although the exact tests used and references to the primary literature are neither discussed nor disclosed. All compounds are NMR and HPLC validated and have their biological activity described. The ApexBio library of protease inhibitors was constructed by cherry picking of compounds that are associated with different proteases. The total number of compounds (825), the quality control certificates and a chart of composition are provided, but little or no information is given about properties such as IC_50_ values or references to filtering protocols. Otava offers no less than 19 different protease libraries, with little information on their design provided. Where this information is available, virtual screening approaches were used to construct the library. These include receptor-based approaches such as pharmacophore and docking protocols to obtain molecules with the predicted activity. No evidence of activity or assays against the selected target is given. The Enamine compound supplier provides a protease-focused library with 117 known protease inhibitors. All of the available compounds were NMR and HPLC validated to ensure quality. The Selleckchem protease-focused library contains 319 small molecules with NMR and HPLC analytical data. It was constructed by manually searching the available literature on protease inhibitors, but no exact literature references are provided. The library has over 140 citations in scientific articles at the time of the writing of this paper. The number of compounds per target molecule is depicted with a graphical representation on the webpage. The compound supplier Asinex also offers protease-focused libraries of compounds, but with no exact details on the design provided to the customer. There are three libraries: the protease library, with 6640 compounds selected from their Signature and BioDesign Libraries; the Protease inhibitors library, with 80 compounds obtained by using screening against a panel of proteases; and the inhibitor library, with 80 compounds obtained by using pharmacophore modeling. ChemDiv offers two libraries: the cysteine and serine protease inhibitors databases. The databases were assembled by using in silico methods and created by following two key approaches: by comparing structural similarity of molecules in the ChemDiv database to molecules with known activity against proteases and by selecting molecules with structural motifs commonly represented in peptidomimetics, with little additional design information provided to the customer. The LifeChemicals protease inhibitor library comprises cystein, serine, aspartic and metallo protease inhibitor libraries. Both ligand- and receptor-based approaches were applied in the construction of this library. The molecules in the library are based on 80,000 reference molecules with a proven activity that were retrieved from the ChEMBL database. Some compounds from the LifeChemicals library were added based on the 2D fingerprint similarity with a Tanimoto comparison cutoff of 85% for serine and cystein proteases and of 75% for metalloproteases. The remaining compounds were selected by using molecular docking into different protease targets and confirming the results by molecular dynamics simulations. PAINS, toxic and reactive compounds were excluded from the library. For each library described, we performed an analysis of the most common molecular descriptors: Molecular Weight (MW), Total Polar Surface Area (TPSA), SlogP, number of hydrogen bond acceptors (HBA), number of hydrogen bond donors (HBD), number of rings present and the number of rotatable bonds (Table 4). Compound retention when passing through each individual filter was assessed, as well (Table 5), with the results for joined filter provided in Figure 9.

### 4.3. Protein–Protein-Interaction-Inhibitor-Focused Libraries

Protein–protein interactions are associated with several crucial biological processes, such as cell growth, proliferation and differentiation. The finding of protein–protein inhibitors represents a complex process due to several factors, including a large interaction surface devoid of clefts and binding pockets. Both large and small molecules have been developed to facilitate this inhibition [89,90]. A filter dubbed the rule of four has been specifically developed for discovering protein–protein inhibitors [69]. We described these targeted libraries mainly with the SARS-CoV-2 Sprot–ACE2 interaction in mind.

**Figure 10:**
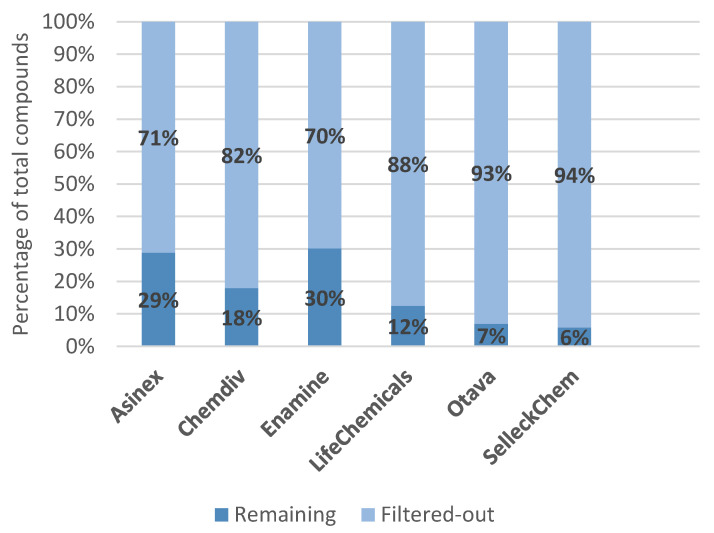
Compound (relative) retention after post-filtering for REOS, PAINS, Aggregators and the Lipinski rule of 5 (one rule break allowed) on protein–protein-interaction-inhibitor-targeted libraries.

The Selleckchem library consists of 188 protein–protein interaction (PPI) inhibitors, which target a vast array of different proteins from epigenetic targets to serine proteases. The library was constructed by filtering the literature on known PPI inhibitors. Certain compounds in the library have been FDA approved, and all of them are NMR and HPLC validated. At the time of writing the site reports 132 publications citing this library. Enamine compound supplier offers a PPI inhibitor library designed by inspecting the available structural data of 20 different protein–protein complexes involved in PPI. Ligand and structure based in silico screening was performed to obtain the 40640 compounds available. The final selection of compounds was based on specific recognition patterns (hot-spot analysis, key amino acids, secondary/tertiary structures), lead-like properties and sp3-rich motifs, passing different rule-based filters such as PAINS and synthetic availability. Asinex provides two different libraries with PPI inhibitors, one with non-macrocyclic compounds and another, which contains small molecules and macrocyclic compounds. The non-macrocyclic library is based upon scaffolds obtained from several α-helix mimetics, that were proven useful for targeting PPIs. Moreover, four pharmacophore models were created based on the available PPI inhibitor structural data and used to determine 3 key-recognition features. This was applied for molecular docking of the α-helix mimetic scaffolds and for the final selection of the molecules in the library. The second library contains molecules that mimic protein structural properties such as β-turn and α-helix, and was assembled using similarity search. LifeChemicals provide four non-overlapping libraries built with different approaches. The first library was devised using machine learning decision trees. By comparing several known PPI inhibitors to non-PPI inhibitors, several descriptors facilitated the model to differentiate between the two. Molecules that fit the descriptors of PPI inhibitors were collected from the entire LifeChemicals HTS compound collection after which medicinal chemistry filters were applied to filter out toxic, reactive and PAINS molecules. The final library consists of 6800 molecules. The second library was constructed using similarity search on the LifeChemicals HTS-library toward a reference set of molecules from three different protein–protein interaction libraries (Timbal, 2P2I and iPPI databases). The similarity search was conducted using the Tanimoto coefficient with the threshold at 85%, the obtained molecules were checked for reactive and inactive compounds. The third library was based upon the rule of four proposed by X.Morelli [69] which states that if the molecules abide several structural rules they belong to the desired chemical space. The fourth and final library was built upon 2D similarity search against molecules from reference databases (Pubmed, ChEMBL) where PPI were confirmed with related assays. The entire LifeChemicals HTS library was filtered using a Tanimoto similarity cutoff of 80% and general medicinal chemistry filters providing the final 14,400 molecules. Otava offers two different PPI inhibitor libraries. The first was designed using a decision tree algorithm, which effectively separates PPI inhibitors from a group non-PPI inhibitor. The second was obtained using Bayesian modeling based on known PPI inhibitors from the TIMBAL database. The Otava database was filtered using a Bayesian similarity score cutoff and general medicinal chemistry filters (Lipinski, MW cutoff). Otava compound supplier also offers two peptidomimetic libraries, which are divided into α-helix peptidomimetics and β-turn peptidomimetics. The α-helical one was assembled using template compounds found in the scientific literature to create training sets for devising models based on different fingerprints such as FCFP6 and ECFP6. The β-turn library was constructed by using pharmacophore screening, where the pharmacophore is based on real β-turns. The second portion of this library was established by using a similarity search of known scaffolds of β-turn peptidomimetics. The ChemDiv library of potential PPI inhibitors contains 210,000 molecules that were collected by using in silico HTS methods that focused on PPI hot spots located on the protein surfaces, while TargetMol provides a small focused library that contains 143 PPI-related compounds with NMR and HPLC supporting analytical data. No further information is provided on how the library was assembled. For each library described, we performed an analysis of the most common molecular descriptors: Molecular Weight (MW), Total Polar Surface Area (TPSA), SlogP, number of hydrogen bond acceptors (HBA), number of hydrogen bond donors (HBD), number of rings present and the number of rotatable bonds (Table 6). Compound retention when passing through each individual filter was assessed, as well (Table 7), with the results for joined filter provided in Figure 10.

## 5. Discussion

**Figure 11:**
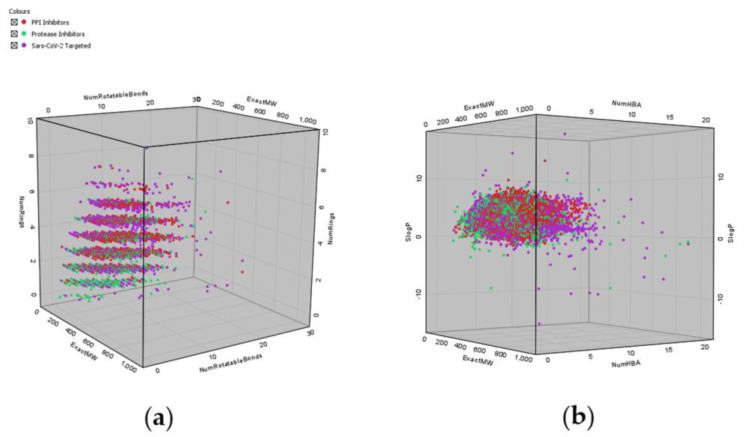
(**a**) Distribution of Molecular Weight (ExactMW), number of rotatable bonds (NumRotatableBonds) and number of rings (NumRings) for all joined libraries represented in a spatial diagram. (**b**) Distribution of Molecular Weight (ExactMW), number of hydrogen bond acceptors (NumHBA) and SlogP for all joined libraries represented in a spatial diagram.

Focusing on commercial availability, one immediately recognizes the abundance of options offered, especially when considering a specific area, such as SARS-CoV-2-targeted libraries and protease-inhibitor-focused or protein–protein-interaction-inhibitor-focused libraries. These libraries provide ready-to-use compounds, and, surprisingly, most commercial vendors also offer the necessary analytical support. Looking at the distribution of molecular mass in these commercial libraries, we can observe a strict focus on drug-like and not lead-like properties. All commercial libraries have the majority of compounds with a Molecular Weight of around 500 g/mol, as shown in Figure 8 and Figure 11. The libraries also follow the misconception of “drug-likeness”, as described by the Lipinski’s rule of five (the rule focuses on oral bioavailability), instead of adopting approaches that would incorporate biological contexts in the form of pharmacodynamics on the focused targets and possibly even pharmacokinetic potentials of designed libraries [47]. Figure 9 displays that no library has compounds with average SlogP values above 3.71 and all libraries follow the rule-of-five guidelines in terms of the number of hydrogen bond donors and acceptors. Interestingly, however, the filters used were not exactly those described in the scientific literature, as Figure 10 depicts compound attrition ranging from 56 to 80% for the REOS, PAINS and Aggregator filters (one-break rule) and from 0.06 to 21% for the Lipinski rule-of-five filter. The attrition rates of each filter are reported in Table 2, with the REOS and Aggregator filters responsible for the highest attrition. Nevertheless, these observations are only focused on the chemical space, since excessive filtering in such small libraries (2000–20,000 compounds) can cause potentially active chemicals to drop out. The main problem with the libraries discussed, in our opinion, is that vendors lack the information on library design, as well as the references to the primary literature. Commercial vendors consistently provide only general information about the screening protocols used to design the target libraries offered and are even less informative about the actual filters used. No references to actual actives are provided when using ligand-based design, with the exception of marketed libraries of known actives that can be referenced post-purchase by the client through databases such as ChEMBL. For structure-based approaches, usually only target or protein classes or a general panel of targets are provided, with reference to methods such as molecular docking. No precise docked receptors or PDB IDs of the targets are available, and no docking protocols or even references to the molecular docking software (or other HTVS software) are provided. This fact is that worrying does not bode well for the use of these libraries in drug design and lends itself to commercial drug companies to focus on and improve. However, another cause for concern is the presence of REOS structures and the lack of focus in the libraries on the design of covalent or non-covalent inhibitors. Often, the functional group composition of the targeted library is not discussed. This should be expected at least from the libraries focused on proteases.

Last but not least, we would like to draw the reader’s attention to the quality of the molecules in the commercial libraries. In our more than 10 years of experience, we have found that the quality of the compounds purchased from commercial suppliers is usually high, with most compounds being characterized by NMR and MS/HRMS analyses after purchase, and their purity being determined to be about 95% or higher by HPLC. However, analytical data are not part of the original catalogue selection, and purity data are not usually available prior to the purchase. Therefore, we recommend that the reader be aware of this fact and even take advantage of asking the commercial vendor for characterization and purity data prior to the purchase (in some cases, NMR, MS and HPLC data can be obtained). Nowadays, vendors can even point out certain availability and quality issues. From the time of purchase, the quality of the supply chain (cold storage, if necessary, and insurance for possible shipping problems) and the emphasis on quality storage after delivery are essential.

## 6. Conclusions

In this paper, we evaluated commercially available targeted libraries, which are often marketed by vendors to the scientific community. Targeted libraries are smaller than general libraries and aim at specific domains or targets, with the aim of reducing the time and computational power required to prepare and process the compounds. We here reported a collection of commercially available SARS-CoV-2-targeted and protease-inhibitor-targeted or protein–protein-interaction-inhibitor-targeted compound libraries suitable for starting the high-throughput virtual screening for COVID-19 research or for the drug design and development of new SARS-CoV-2 antivirals. When we examined the processes behind the design of these libraries and evaluated them by using filtering and descriptor analysis, we could see that the library design is not transparent and that the exact steps on the way to focused libraries are not provided by the commercial vendors, nor are the references to the primary literature included. The authors also acknowledge that the state-of-the-art in commercial focused or targeted libraries goes well beyond the limited scope of COVID-19 reported in this paper. We, therefore, encourage commercial and other providers of focused and targeted libraries to address this lack of data for the initial virtual screening steps that these libraries are designed to support. Readers should be cautious when using targeted libraries and ensure that they are familiar with the design processes used, as compound selection is critical for the success of drug discovery efforts. If the respected reader would like to develop his/her own focused libraries, the article provides step-by-step guidelines and highlights critical aspects to consider for the in silico library design in medicinal chemistry.

## Figures and Tables

**Table 1:** Essential filters for efficient library design in medicinal chemistry.

Name	Features/Cutoffs	Developer/ Reference
PAINS	Removal of frequent hitters (promiscuous compounds) in HTS assays	Cancer Therapeutics-CRC P/L [64]
REOS	Set of rules and of functional group filters for the removal of problematic structures dubbed REOS (Rapid elimination of swill) Maintaining compounds using the following cutoffs: H-bond donor ≤ 5, H-bond acceptors ≤ 10, −2 ≤ formal charge ≤ +2, number of rotatable bonds ≤ 8, 200 ≤ Molecular weight ≤ 500, 20 ≤ number of heavy atoms ≤ 50, −2 ≤ logP ≤ 5	Vertex [65,66]
Aggregators	Tanimoto coefficient similarity search to a database of known aggregators	Irwin et al. [52]
Ely Lilly rules	Hybrid method of physiochemical and functional group filters for identification of promiscuous compounds	Bruns at Ely Lilly [67]
Lipinski (Rule of 5)	A set of rules for drug-likeness: Molecular Weight ≤ 500, logP ≤ 5, H-bond donors ≤ 5, H-bond acceptors ≤ 10	Lipinski at Pfizer [46]
Rule of 3	A set of cutoff rules for lead-like discovery: Molecular Weight ≤ 300, logP ≤ 3, H-bond donor ≤ 3, H-bond acceptors ≤ 3	Congreve et al. [68]
Rule of 4	A set of rules derived from protein–protein interaction inhibitors: Molecular Weight ≥ 400, logP ≥ 4, number of rings ≥ 4, H-bond acceptors ≥ 4	Morelli [69]
Oprea Lead-like and drug-like	A set of rules based on the lead-like vs. drug-like comparison: Molecular Weight < 450, −3.5 ≤ logP < 4.5, −4 ≤ logD ≤ 4, number of rings ≤ 4, nonterminal single bonds ≤ 10, H-bond donor ≤ 5, H-bond acceptor ≤ 8	Oprea group [70]
Ghose	A set of rules for drug-likeness with cutoffs: 180 ≤ Molecular Weight ≤ 480, 40 ≤ molecular refractivity ≤ 130, −0.4 ≤ logP ≤ 5.6, 20 ≤ number of atoms ≤ 70	Ghose et al. [71]
Veber	Two rules to meet the criteria for drug-likeness: rotatable bonds ≤ 10, Polar Surface Area ≤ 140 Å^2^	Veber et al [72]
Lee	Physiochemical properties of highly-drug like molecules: Mean Molecular Weight = 356 Mean logP = 2.1	Lee at Hoffman-La Roche [73]
van de Waterbeemd	Physiochemical properties for permeability and blood brain barrier permeability: Molecular Weight ≤ 450, Polar Surface Area ≤ 90 Å^2^	van de Waterbeemd [74]
Mozzicconacci	Set of rules to filter for drug-like molecules: Rotatable bonds ≤ 15, rings ≤ 6, oxygen atoms ≥ 1, nitrogen atoms ≥ 1, halogen atoms ≤ 7	Mozziconacci [75]
Fichert	Rules for permeability based on a small drug set: Molecular Weight ≤ 500, 0 ≤ logD ≤ 3	Fichert et al. [76]
Muegge method	Bioavailability prediction rules dubbed Muegge method: 200 ≤ MW ≤ 600, −2 ≤ logP ≤ 5, PSA ≤ 150 Å^2^, number of rings ≤ 7, number of carbons ≥ 4, number of heteroatoms > 1, Number of rotatable bonds ≤ 15, H-bond acceptors ≤ 10, H-bond donors ≤ 5	Muegge [77]
Egan	Set of rules designed to predict bioavailability: logP ≤ 5.88, PSA ≤ 131.6 Å^2^	Egan et al. [78]
Murcko filter	Set of rules derived from the rule of 5 coupled with 1D and 2D descriptor analysis to determine central nervous system activity. MW 200–400, 0 ≤ logP ≤ 5.2, H-bond acceptors ≤ 4, H-bond donor ≤ 3, rotatable bonds ≤ 7	Ajay et al. [79]

**Table 2:** Analysis of the most important descriptors for the SARS-CoV-2-targeted library.

Database Name	No. of Compounds	MW	TPSA	SlogP	HBA	HBD	No. of Rings	No. of Rotatable Bonds
Chembridge	16,777	391.5 ± 62	80 ± 26	3.2 ± 1.5	4.9 ± 1.7	1.4 ± 1.0	4.3 ± 0.8	3.9 ± 1.4
Enamine	16,800	362 ± 61	79.3 ± 21	2.7 ± 1.2	4.5 ± 1.4	1.58 ± 0.8	3.1 ± 0.8	5.1 ± 1.8
LifeChemical	7311	404 ± 75	84.8 ± 23	3.1 ± 1.4	5.8 ± 1.8	1.4 ± 0.9	3.6 ± 0.9	5.6 ± 1.9
Otava	9018	383 ± 56	77.3 ± 20	3.7 ± 1.0	5.2 ± 1.5	1 ± 0.8	3.9 ± 0.8	4.0 ± 1.5
TargetMol	2448	460 ± 211	110 ± 151	2.6 ± 4.6	6.6 ± 4.0	2.2 ± 3.2	3.6 ± 1.5	7.5 ± 4.8
Joined SARS-CoV-2-Targeted Library	52,354	385 ± 79	81 ± 40	3.1 ± 1.7	5.0 ± 1.9	1.4 ± 1.1	3.7 ± 1.0	4.7 ± 2.1

**Table 3:** Analysis of the attrition rate of various filters for the SARS-CoV-2-targeted library.

	Chembridge	Enamine	LifeChemicals	Otava	TargetMol
Unfiltered	16,777	16,800	7311	9018	2448
Isolated filter:	Number of filtered out compounds (%)				
REOS	1160 (7%)	4565 (27%)	1414 (19%)	1486 (17%)	858 (35%)
PAINS	454 (3%)	267 (2%)	11 (~0%)	430 (5%)	248 (10%)
Aggregators	9053 (54%)	6702 (40%)	4002 (55%)	6784 (75%)	1445 (60%)
Lipinski Ro5	258 (2%)	193 (1%)	380 (5%)	5 (~0%)	522 (21%)
All filters	10,000 (60%)	9412 (56%)	4937 (68%)	7202 (80%)	1887 (77%)

**Table 4:** Analysis of the most important descriptors for the protease-inhibitor-targeted library.

Database Name	No. of Compounds	MW	TPSA	SlogP	HBA	HBD	No. of Rings	No. of Rotatable Bonds
ApexBio	824	348 ± 181	96 ± 59	1.9 ± 2.8	5.0 ± 3.2	2.5 ± 2.0	2.5 ± 1.7	5.2 ± 4.5
Asinex	6640	383 ± 34	79 ± 18	2.9 ± 1.0	5.3 ± 1.3	0.9 ± 0.7	3.7 ± 0.6	4.7 ± 1.5
Chemdiv	41,801	406 ± 63	74 ± 20	3.6 ± 1.2	5.0 ± 1.6	1.1 ± 0.7	3.7 ± 0.8	5.4 ± 1.8
Enamine	117	336 ± 167	90 ± 58	2.2 ± 2	4.4 ± 2.6	2.0 ± 1.8	2.5 ± 1.5	4.5 ± 4.4
LifeChemicals	25,535	390 ± 70	81 ± 22	3.0 ± 1.4	5.3 ± 1.7	1.0 ± 0.7	3.3 ± 0.9	4.9 ± 1.9
Otava	8034	352 ± 71	79 ± 23	3.0 ± 1.2	4.6 ± 1.6	1.4 ± 0.9	3.2 ± 1.1	4.5 ± 1.8
SelleckChem	227	409 ± 168	106 ± 52	2.4 ± 2	5.45 ± 2.6	2.4 ± 1.7	2.9 ± 1.7	6.2 ± 4.4
TargetMol	295	410 ± 183	107 ± 60	2.4 ± 2.2	5.6 ± 3.1	2.5 ± 2.0	3.0 ± 1.8	5.9 ± 4.5
Joined Protease Inhibitor Databases	83,473	394 ± 70	77 ± 23	3.3 ± 1.3	5.0 ± 1.7	1.1 ± 0.8	3.5 ± 0.9	5.1 ± 1.9

**Table 5:** Analysis of the attrition rate of various filters for the protease-inhibitor-targeted library.

	ApexBio	Asinex	Chemdiv	Enamine	LifeChemicals	Otava	SelleckChem	TargetMol
Unfiltered	824	6640	41,801	117	25,535	8034	227	295
Isolated filter:	Number of filtered out compounds (%)							
REOS	397(48%)	273(4%)	2151(5%)	54(46%)	3583(14%)	1025(13%)	110(48%)	146(49%)
PAINS	58(7%)	129(2%)	1060(3%)	7(6%)	430(2%)	307(4%)	7(3%)	16(5%)
Aggregators	294(36%)	3254(49%)	29,313(70%)	36(31%)	14,059(55%)	4216(52%)	88(39%)	118(40%)
Lipinski Ro5	113(14%)	0(0%)	1045(2%)	11(9%)	409(2%)	2(~0%)	37(16%)	55(19%)
All filters	743(90%)	5019(76%)	37,137(89%)	106(91%)	23,215(91%)	6987(87%)	208(92%)	272(92%)

**Table 6:** Analysis of the most important descriptors for the protein–protein-interaction-inhibitor-targeted library.

Database Name	No. of Compounds	MW	TPSA	SlogP	HBA	HBD	No. of Rings	No. of Rotatable Bonds
Asinex	11,439	386 ± 53	70 ± 19	3.1 ± 1.2	4.9 ± 1.3	0.8 ± 0.6	3.7 ± 0.7	4.9 ± 1.5
Chemdiv	212,906	408 ± 61	72 ± 20	3.5 ± 1.3	5.0 ± 1.6	0.8 ± 0.7	3.9 ± 0.8	5.1 ± 1.9
Enamine	40,640	357 ± 49	70 ± 17	2.8 ± 0.9	4.7 ± 1.3	1 ± 0.7	3.5 ± 0.7	4.3 ± 1.6
LifeChemicals	36,426	393 ± 81	77 ± 22	3.3 ± 1.1	5.4 ± 1.8	1.0 ± 0.7	3.6 ± 1.0	5.3 ± 2.2
Otava	3849	437 ± 71	86 ± 25	3.8 ± 1.6	5.4 ± 1.6	1.4 ± 1.0	3.9 ± 1.1	6.4 ± 2.4
SelleckChem	188	472 ± 183	101 ± 66	3.6 ± 2.3	6.3 ± 3.7	2.1 ± 1.7	3.8 ± 1.5	6.2 ± 3.9
Joined PPI databases	305,448	400 ± 65	73 ± 20	3.3 ± 1.2	5.0 ± 1.6	0.9 ± 0.7	3.8 ± 0.8	5.0 ± 1.9

**Table 7:** Analysis of the attrition rate of various filters for the protein–protein interaction inhibitor library.

	Asinex	Chemdiv	Enamine	LifeChemicals	Otava	SelleckChem
Unfiltered	11,439	212,906	40,640	36,426	3849	188
Isolated filter:	Number of filtered out compounds (%)					
REOS	148(1%)	12,546(6%)	970(2%)	3191(9%)	686(18%)	87(47%)
PAINS	18(~0%)	3891(2%)	239(1%)	547(2%)	164(4%)	22(12%)
Aggregators	6579(58%)	132,015(62%)	17,499(43%)	22,721(38%)	2682(70%)	126(67%)
Lipinski Ro5	26(~0%)	5936(3%)	16(~0%)	814(89%)	424(11%)	47(25%)
All filters	8137(71%)	174,713(82%)	28,386(70%)	31,886(88%)	3581(93%)	177(94%)

## Supplementary Materials

The following are available online at https://www.mdpi.com/article/10.3390/ijms23010393/s1.

## Abbreviations

HTVS	high-throughput virtual screening
CADD	computer-aided drug design
QSAR	quantitative structure-activity relation
PBVS	pharmacophore-based virtual screening
DVBS	docking-based virtual screening
SDF	Structure Data Format
MOL	MDL Molfile
PPI	protein–protein interaction
MW	Molecular Weight
TPSA	Total Polar Surface Area
HBA	number of hydrogen bond acceptors
HBD	number of hydrogen bond donors
