# Supplementary material for: Commercial SARS-CoV-2 Targeted, Protease Inhibitor Focused and Protein–Protein Interaction Inhibitor Focused Molecular Libraries for Virtual Screening and Drug Design
Source: Int J Mol Sci. 2021 Dec 30;23(1):393. doi: 10.3390/ijms23010393 (PMC8745317; doi:10.3390/ijms23010393)
Supplement: Supplementary file 1 [file ijms-23-00393-s001.zip › ijms-1522232-supplementary/kralj_commercial_libraries_supplementary.pdf]

Supplementary Materials

for

Commercial SARS-CoV-2 Targeted, Protease Inhibitor Focused and Protein–Protein Interaction Inhibitor Focused Molecular Libraries for Virtual Screening and Drug Design

Sebastjan Kralj [1], Marko Jukič [1, 2] and Urban Bren [1,2]

[1] Laboratory of Physical Chemistry and Chemical Thermodynamics, Faculty of Chemistry and Chemical Engineering, University of Maribor, Smetanova ulica 17, SI-2000 Maribor, Slovenia

[2] Faculty of Mathematics, Natural Sciences and Information Technologies, University of Primorska, Glagoljaška 8, SI-6000 Koper, Slovenia

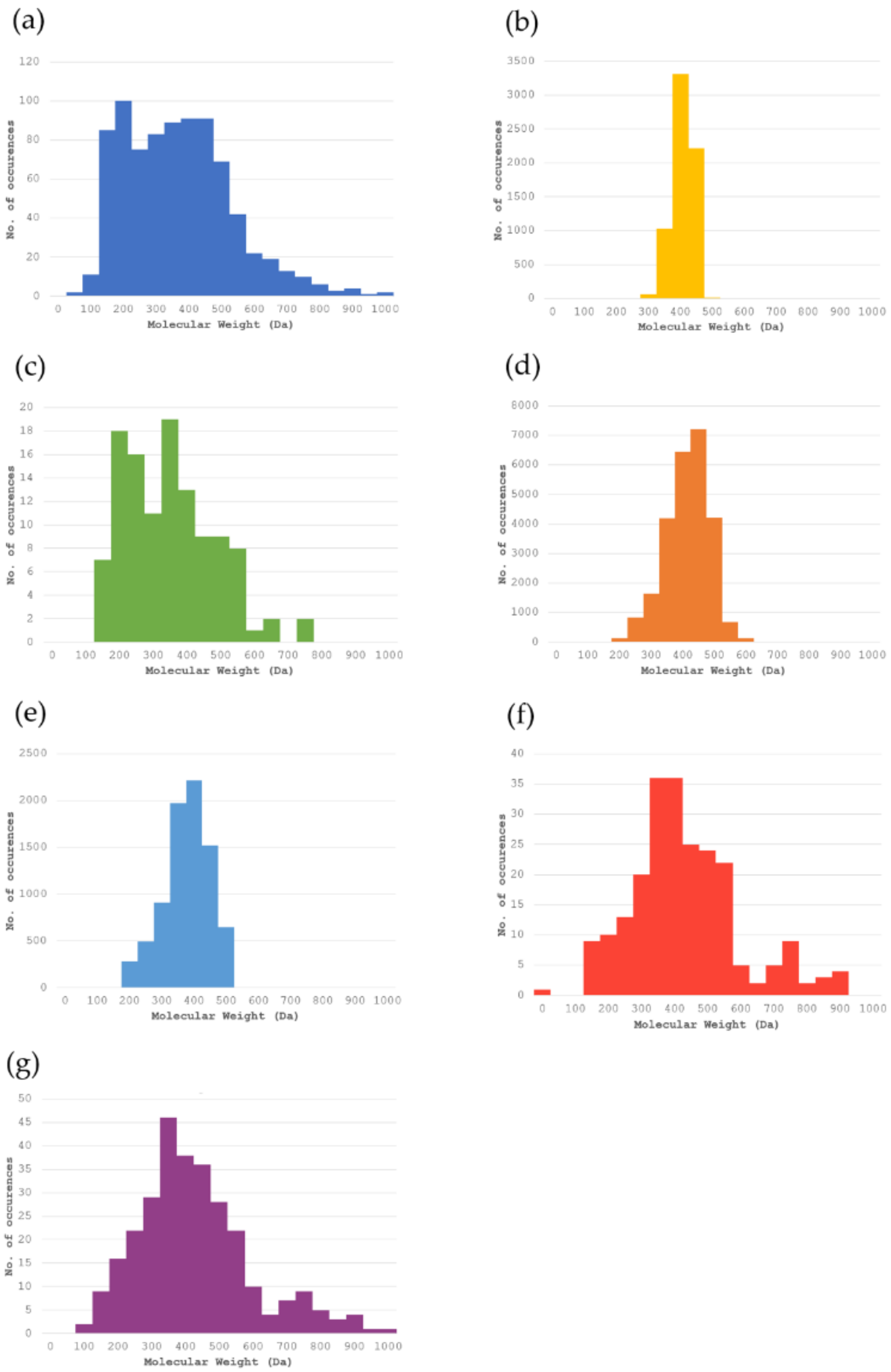

Figure S1: Distribution of Molecular Weight for Protease Inhibitor libraries **(a)** Apex Bio; **(b)** Asinex; **(c)** Enamine; **(d)** LifeChemicals; **(e)** Otava; **(f)** SelleckChem; **(g)** TargetMol.

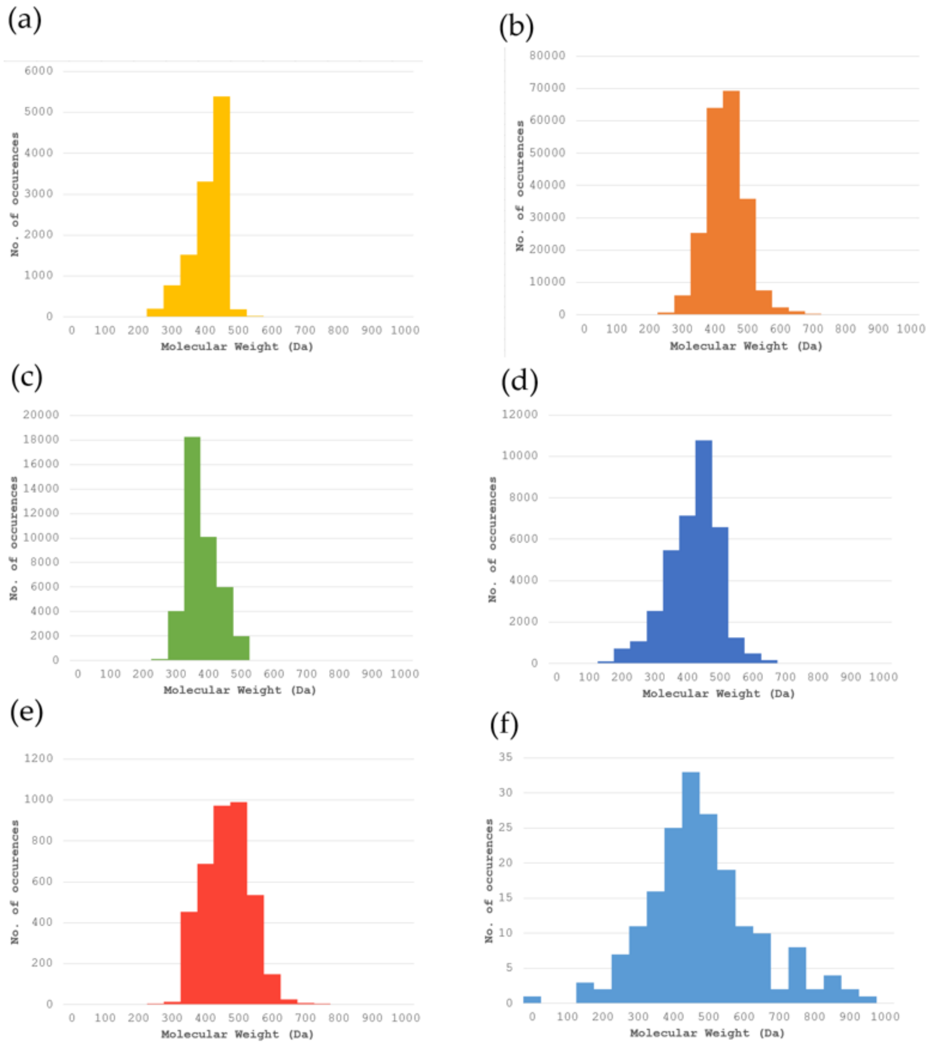

Figure S2: Distribution of Molecular Weight for Protein-Protein Interaction Inhibitor libraries **(a)** Asinex ; **(b)** Chemdiv; **(c)** Enamine; **(d)** LifeChemicals; **(e)** Otava; **(f)** SelleckChem.

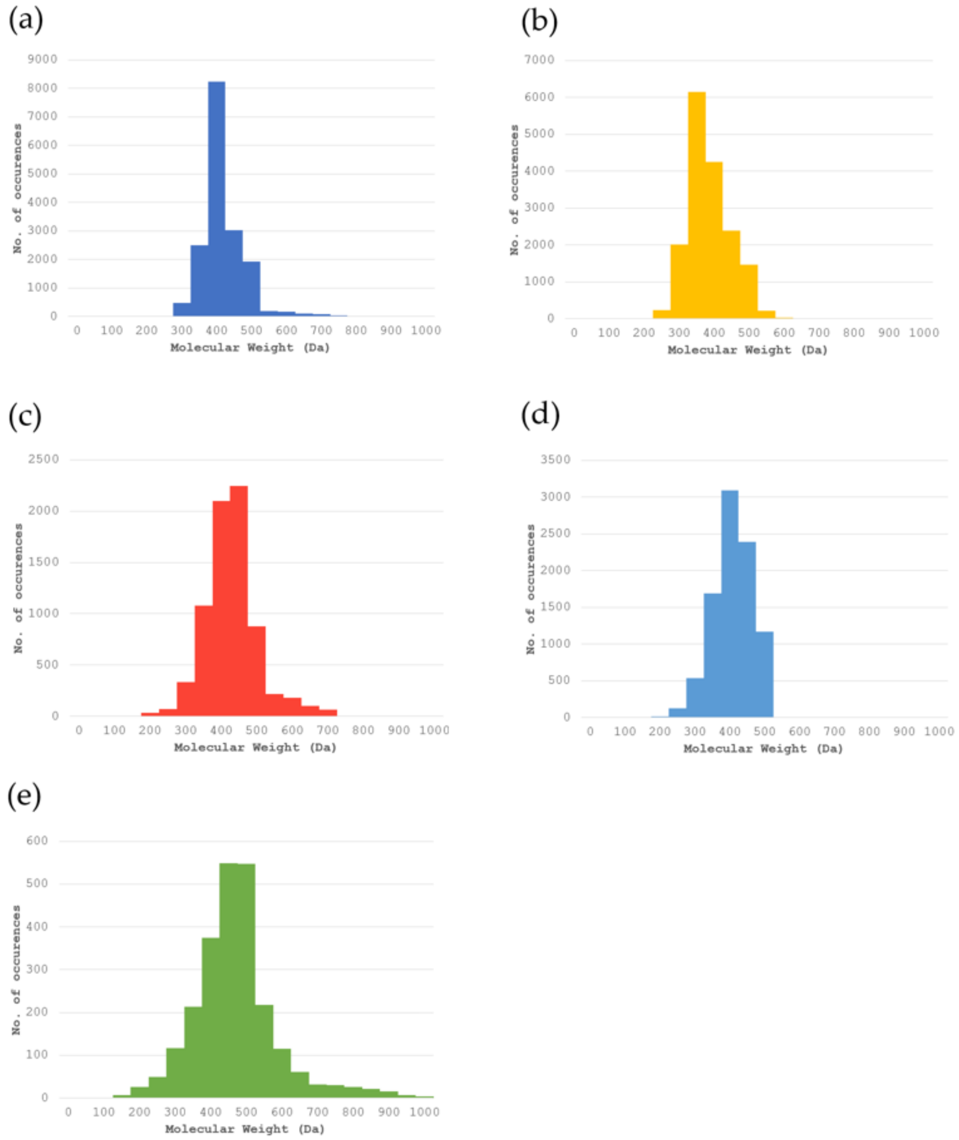

Figure S3: Distribution of Molecular Weight for SARS-CoV-2 target libraries **(a)** Chembridge; **(b)**Enamine; **(c)** LifeChemicals; **(d)** Otava; **(e)** TargetMol.

Supplementary Table S1. Database name and web location

**Database name**	**Location:**
Enamine - Coronavirus library	https://enamine.net/compound-libraries/targeted-libraries/coronavirus-library
Otava - SARS-CoV-2 targeted libraries	https://otavachemicals.com/products/targeted-libraries-and-focused-libraries/sars-cov-2
Chembridge – Coronavirus screening library	https://chembridge.com/screening_libraries/targeted_libraries/coronavirus-library/index.php
LifeChemicals – Coronavirus screening library	https://lifechemicals.com/news/new-coronavirus-screening-libraries
TargetMol- SARS-CoV-2 library	https://www.targetmol.com/library-collection-2/COVID-19-Related-Libraries
TargetMol – Protease inhibitor Library	https://www.targetmol.com/compound-library/Protease%20Inhibitor%20Library
ApexBio- DiscoveryProbe™ Protease inhibitor library	https://www.apexbt.com/discoveryprobetm-protease-inhibitor-library.html

Otava – Protease targeted library	https://www.otavachemicals.com/products/targeted-libraries-and-focused-libraries/protease-targeted-libraries
Enamine – Protease targeted library	https://enamine.net/compound-collections/bioreference-compounds/protease-inhibitors
Selleckchem- Protease inhibitor library	https://www.selleckchem.com/screening/protease-inhibitor-library.html
Asinex- Protease Inhibitors	https://www.asinex.com/protease/
Chemdiv- Cysteine protease inhibitor library	https://www.chemdiv.com/cysteine-proteases-inhibitors-library/
Chemdiv- Serine protease inhibitor library	https://www.chemdiv.com/serine-proteases-inhibitors-library/
LifeChemicals- Protease targeted library	https://lifechemicals.com/screening-libraries/targeted-and-focused-screening-libraries/protease-screening-libraries/protease-targeted-library
Selleckchem- Protein-protein interaction inhibitor library	https://www.selleckchem.com/screening/protein-protein-interaction-inhibitor-library.html
Enamine- PPI library	https://enamine.net/compound-libraries/targeted-libraries/ppi-library
Asinex- Protein-protein interaction	https://www.asinex.com/ppi/
LifeChemicals- PPI screening library	https://lifechemicals.com/screening-libraries/targeted-and-focused-screening-libraries/ppi-libraries/
Otava – iPPI focused library	https://otavachemicals.com/products/targeted-libraries-and-focused-libraries/protein-protein-interaction
Chemdiv- Protein-protein interaction library	https://www.chemdiv.com/protein-protein-interaction-ppi-library/
TargetMol – PPI inhibitors library	https://www.targetmol.com/compound-library/PPI%20Inhibitors%20Library

A comprehensive collection of commercial targeted or focused libraries is in supplied supplementary MS Excel file named: commercial_targeted_or_focused_libraries_collection.xlsx.
